# Regulation of *PTEN* expression by the SWI/SNF chromatin-remodelling protein BRG1 in human colorectal carcinoma cells

**DOI:** 10.1038/sj.bjc.6606018

**Published:** 2010-11-23

**Authors:** T Watanabe, S Semba, H Yokozaki

**Affiliations:** 1 Division of Pathology, Department of Pathology, Kobe University Graduate School of Medicine, 7-5-1 Kusunoki-cho, Chuo-ku, Kobe 650-0017, Japan

**Keywords:** BRG1, BRM, PI3K–Akt signalling pathway, PTEN, cyclin D1, colorectal carcinoma

## Abstract

**Background:**

Aberrant expression of Brahma-related gene-1 (BRG1), a core component of the SWI/SNF chromatin-remodelling complex, has been implicated in cancer development; however, the biological significance of BRG1 in colorectal carcinoma (CRC) remains unknown.

**Methods:**

In CRC tissues, expression of BRG1 and Brahma (BRM) was investigated immunohistochemically. Colorectal carcinoma-derived DLD-1 cells were used for knockdown of BRG1 and PTEN with small interfering RNA (siRNA) and transduction of Akt. Complementary DNA (cDNA) microarray analysis was performed to explore the genes affected by BRG1.

**Results:**

Expression of BRG1, but not BRM, was frequently elevated in CRC specimens, and knockdown of BRG1 suppressed cell proliferation of DLD-1 cells. By cDNA microarray, we determined that PTEN expression was negatively regulated by BRG1 in DLD-1 cells, which subsequently influenced the cyclin D1 levels via the phosphoinositide 3-OH kinase (PI3K)–Akt signalling pathway. The interplay of BRG1 on cyclin D1 expression was confirmed by the introduction of Akt and knockdown of PTEN in the *BRG1* siRNA-transduced DLD-1 cells. Interestingly, this positive correlation between BRG1 and cyclin D1 expression was also observed in CRC specimens.

**Conclusion:**

Brahma-related gene-1 has an important role in the process of CRC development by activating the PI3K–Akt signalling pathway and resultant upregulation of cyclin D1 levels.

Chromatin is actively remodelled during development, as indicated by the observations that the same genetic locus in different tissues varies in its sensitivity to DNase I and restriction enzymes (Weintraub and Groudine, 1976; McGhee *et al*, 1981). Chromatin remodelling of certain genes appears to precede the transcriptional activation of the genes, suggesting that chromatin remodelling may occur in anticipation of developmental transitions (Siebenlist *et al*, 1986). The SWI/SNF chromatin-remodelling complex is a multisubunit complex first identified in yeast and highly conserved among eukaryotes (Kingston and Narlikar, 1999; Wade and Wolffe, 1999). The mammalian SWI/SNF complex mediates ATP-dependent chromatin remodelling processes that are critical for transcriptional regulation by remodelling of nucleosomes, control of cellular processes and involvement in DNA repair, proliferation and differentiation (Roberts and Orkin, 2004; Dinant *et al*, 2008; Reisman *et al*, 2009). The SWI/SNF complex contains 9–12 different subunits that assemble into at least three separate complexes containing either single Brahma-related gene-1 (BRG1) or Brahma (BRM) as the ATPase subunit (Wang *et al*, 1996). The BRG1 and BRM possess highly conserved structures, with a sequence identity of 75% in humans, and their enzymatic properties are quite similar (Khavari *et al*, 1993; Chiba *et al*, 1994). Despite the fact that these subunits are interchangeable (Phelan *et al*, 1999), the mechanism by which the functions of BRG1 and BRM are distinguished in the SWI/SNF complex is currently poorly understood.

Brahma-related gene-1 has been reported to affect cell growth and to interact with the regulatory proteins involved in cellular proliferation in *in vitro* studies (Muchardt and Yaniv, 2001). Transduction of BRG1 into BRG1- and BRM-negative cells inhibited cell proliferation through altered expression of retinoblastoma (Rb) family members (Dunaief *et al*, 1994; Strober *et al*, 1996; Dahiya *et al*, 2000). In breast carcinoma cells, the induction of cell cycle arrest by reintroduction of BRG1 was accounted for by the downregulation of *cyclin E* and upregulation of cyclin-dependent kinase inhibitors *p21* and *p15* expression (Hendricks *et al*, 2004). In addition, BRG1 protein directly interacts with BRCA1 tumour suppressor and subsequently stimulates transcriptional activity of the p53 protein (Bochar *et al*, 2000). Thus, evidence has accumulated that supports the tumour-suppressive effects of BRG1 in human cancers. However, increased expression of BRG1 was oppositely oncogenic and indispensable for transformation of human cervical, rhabdoid and colon cancer cells: BRG1 permitted cancer cell proliferation in cooperation with the histone acetyl transferase protein, CREB-binding protein, to suppress p53 activity (Naidu *et al*, 2009). Thus, BRG1 may possibly be involved in biological processes that accelerate cell cycle progression and cell proliferation.

In human cancers, aberrant expressions of BRG1 and BRM have been documented in the development of tumours, including those of the stomach (Sentani *et al*, 2001; Yamamichi *et al*, 2007), lung (Reisman *et al*, 2003), prostate (Sun *et al*, 2007) and melanocytes (Lin *et al*, 2010); nevertheless, there is a major discrepancy in the biological significance of BRG1. The *BRG1* gene deletion or mutation was found in SW13 adrenocortical carcinoma cells and PANC-1 pancreatic adenocarcinoma cells (Reisman *et al*, 2003). Also, ∼10% of primary lung cancers showed a concomitant loss of BRG1 and BRM expression, which was closely correlated with poor prognosis (Reisman *et al*, 2002). On the other hand, increased expressions of BRG1 and BRM were associated with development and progression of prostate cancer (Sun *et al*, 2007), cutaneous melanoma (Lin *et al*, 2010) and gastric carcinoma (Sentani *et al*, 2001). These findings indicate the possibility that the biological significance of these SWI/SNF chromatin remodelling complex molecules during the pathogenesis of human cancer differs according to cell and/or tissue types.

In this study, we investigated the pathological significance and underlying mechanisms of BRG1 and BRM in human colorectal carcinoma (CRC). We performed immunostaining of BRG1 and BRM in primary CRC specimens as well as their adjacent normal mucosa and adenoma. Knockdown of BRG1 by RNA interference was conducted for cell growth test and gene expression profiling experiment.

## Materials and methods

### Cell lines and tissue samples

Human CRC cell lines DLD-1, SW480, HCT116, LoVo and SW620 were obtained from the American Type Culture Collection (Manassas, VA, USA). Cells were cultured in RPMI-1640 medium containing 10% fetal bovine serum. Cells were treated with phosphoinositide 3-OH kinase (PI3K) inhibitor LY294002 (Sigma, St Louis, MO, USA) dissolved in DMSO at a final concentration of 20 *μ*M. A total of 31 cases of human CRCs and adenomas surgically or endoscopically removed at Kobe University Hospital (Kobe, Japan) were employed. Informed consent was obtained from all patients and the study was approved by the institutional review committee of the Kobe University. Histological examination was performed according to *the Japanese Classification of Colorectal Carcinoma* (Japanese Society for Cancer Colon and Rectum, 1998) along with the International Union Against Cancer classification (Sobin and Wittekind, 1997).

### Immunohistochemistry and immunofluorescence

Immunohistochemistry was performed using the Labelled StreptAvidin-Biotin kit (Dako, Copenhagen, Denmark). Antibodies against BRG1 (Santa Cruz, Santa Cruz, CA, USA), BRM (Abcam, Cambridge, MA, USA), cyclin D1 (Cell Signaling, Beverly, MA, USA), PTEN (Cell Signaling) and phospho-Akt (Ser473) (p-Akt, Cell Signaling) were used. Sections were incubated with biotinylated goat anti-mouse/rabbit IgGs, and streptavidin conjugated to horseradish peroxidase (HRP) was used to immerse with 3,3-diaminobenzidine tetrahydrochloride. Immunoreactivities of BRG1, BRM, cyclin D1, PTEN and p-Akt were graded according to the staining intensity in individual cells: (−), <30% of tumour cells showed weak immunoreactivities; and (+), >30% of tumour cells showed intense immunoreactivities. For immunofluorescence, cells were grown on glass coverslips and then fixed with 1% formaldehyde. Antibodies against BRG1, BRM, E-cadherin (Santa Cruz), *β*-catenin (Cell Signaling), p-Akt, phospho-GSK-3*β* (Ser9) (p-GSK-3*β*, Cell Signaling) and cyclin D1 were used. Staining patterns were visualised with Cy2- or Cy3-conjugated antibody against rabbit/mouse IgGs (GE Healthcare Biosciences, Little Chalfont Buckinghamshire, UK). The nuclei were stained with 4,6-diamidino-2-phenylindole (DAPI).

### RNA interference, gene transfection, cell growth test and flow cytometry

Cells were plated at a density of 1 × 10^5^ cells and treated with *BRG1* or negative control Stealth RNAi small interfering RNA (siRNA) duplex oligoribonucleotide at a final concentration of 20 nM (Invitrogen, Carlsbad, CA, USA) using Lipofectamine RNAi MAX (Invitrogen). We also used *PTEN* Stealth RNAi siRNA (Invitrogen). Wild-type *Akt1* expression vector (p-Akt1; Upstate, Lake Placid, NY, USA) were transfected into DLD-1 cells using Lipofetamine 2000 (Invitrogen). For cell growth test, cells were plated at a density of 5.0 × 10^4^. We counted the number of the viable cells with cell counting chamber. To analyse cellular DNA content, DLD-1 cells were collected and fixed in 70% methanol, treated with RNase A and stained with propidium iodide. The analysis was performed with a FACS Calibur cytometer (BD Biosciences, San Jose, CA, USA). Cell viability was evaluated from the population of cells in the subG_1_ DNA content.

### Western blot

The cells were lysed in a buffer containing 50 mM Tris-HCl (pH 7.4), 125 mM NaCl, 0.1% Triton X-100 and 5 mM EDTA containing 1% protease inhibitor cocktail (Sigma). Proteins (20 *μ*g) were separated by sodium dodecyl sulphate–polyacrylamide gel electrophoresis followed by electrotransfer onto Hybond C membrane (Millipore, Bedford, MA, USA). Primary antibodies against BRG1, BRM, PTEN, Akt (Cell Signaling), p-Akt, p-GSK-3*β*, GSK-3*β* (Cell Signaling), cyclin D1 and phospho-cyclin D1 at Thr286 (p-cyclin D1; Cell Signaling) were used. Anti-*β*-actin antibody (Sigma) was used for a loading control. After blotting with primary antibodies, HRP-conjugated anti-mouse/rabbit IgGs (1 : 1000 dilution; GE Healthcare Biosciences) were used as secondary antibodies. The signals were visualised with enhanced chemiluminescence.

### cDNA microarray

Total RNAs were extracted from the *BRG1* siRNA- and control siRNA-treated DLD-1 cells using the RNeasy kit (Qiagen, Hilden, Germany). Double-stranded cDNA was synthesised from 500 ng of total RNA Moloney murine leukaemia virus-reverse transcriptase (Agilent, Palo Alto, CA, USA) and poly dT primer incorporating the T7 promoter. Cy5-sample cRNA and Cy3-common reference cRNA were generated and hybridised to a Whole Human Genome oligo DNA microarray kit (Agilent Technologies), which was scanned using an Agilent DNA microarray scanner (Agilent), as described previously (Takeuchi *et al*, 2006). After data normalisation, significance analysis of microarray plot analysis was performed and significantly altered genes were identified in accordance with the manufacture's instructions (http://chem.agilent.com).

### Quantitative real-time RT–PCR (qRT–PCR)

First-strand cDNA was synthesised using ReverTra Ace (Toyobo, Tokyo, Japan). In order to analyse the expression level of each mRNA, real-time quantitative PCR was performed using the ABI StepOne Realtime PCR system (Applied Biosystems, Foster City, CA, USA). Gene-specific primers were designed using the Primer Express software (Applied Biosystems). The primer sequences were as follows: *PTEN*: 5′-GACATTATGACACCGCCAAA-3′/5′-AAGTTCTAGCTGTGGTGGGTTATG-3′ and *cyclin D1*: 5′-GGGAGGGCAGTTTTCTAATGGA-3′/5′-CACCACAGTGGCCCACACT-3′. RT–PCR amplification was performed after 30 s of denaturation at 95 °C, and 40 cycles of PCR were performed at 95 °C for 5 s and 60 °C for 30 s. We confirmed that a band of single amplicon was detected in each real-time PCR reaction by the following electrophoresis. The *C*_T_ values were determined by plotting the observed fluorescence against the cycle number. Each *C*_T_ value was analysed using the comparative threshold cycle method and normalised to the *C*_T_ values of *cyclophilin A*. The relative gene expression levels were estimated using the following formula: relative expression=2^−(CT[*target gene*]−CT[*cyclophilin A*])^.

## Results

### Increased levels of BRG1 expression in human CRC cases

**Figure 1:**
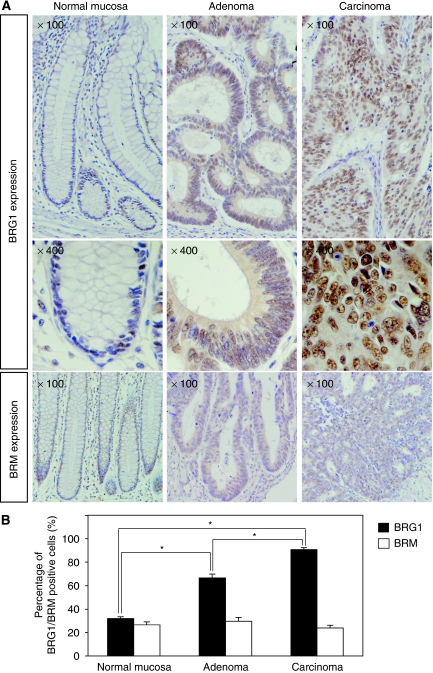
The SWI/SNF chromatin-remodelling BRG1 and BRM expression in human CRC tissues. (**A**) Immunohistochemical results of BRG1 and BRM expression in the representative normal mucosa, adenoma and adenocarcinoma of the colorectum. (**B**) Average percentages of BRG1- and BRM-positive cells. ^*^P<0.05, Student's *t*-test.

To investigate the role of BRG1 and BRM during the pathogenesis of CRC, we first investigated the expressions of BRG1 and BRM in human CRC as well as normal colorectal mucosa and adenoma. In normal mucosa, weak immunoreactivities of BRG1 and BRM in the nuclei were detected, particularly in the cells located at the proliferative zone of crypts (Figure 1A). In CRC tissues, the BRG1 expression levels were dramatically increased, clearly indicating upregulation in comparison with those of adenoma (Figure 1A). However, no significant elevation of BRM levels was found in the same tissue samples (Figure 1A). We evaluated the average ratio of BRG1-positive cells in normal colorectal mucosa, adenoma and CRC as well as that of BRM-positive cells, and found that the average ratios of BRG1-positive cells in normal colorectal mucosa, adenoma and CRC were 31.9±1.6, 66.5±3.3, and 90.6±1.8%, respectively (*P*<0.05, Student's *t*-test, Figure 1B). However, there was no significant difference in the ratios of BRM-positive cells among normal colorectal mucosa, adenoma and CRC (Figure 1B).

### BRG1 knockdown reduces cell proliferation and induces morphological changes

**Figure 2:**
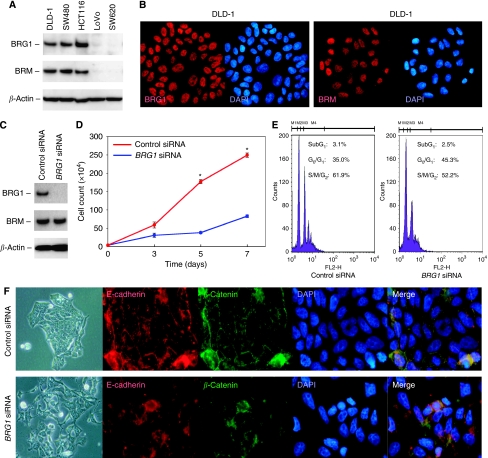
Effects of knockdown of BRG1 in human CRC cell lines. (**A**) Expressions of BRG1 and BRM in CRC cell lines. *β*-Actin was used as a loading control. (**B**) Nuclear localisation of BRG1 and BRM protein in DLD-1 cells. (**C**) Silencing of BRG1 by transduction of *BRG1* siRNA into DLD-1 cells. The negative control siRNA was also transduced. (**D**) Results of cell growth test. ^*^P<0.05, Student's *t*-test. (**E**) Results of cell cycle analysis. The percentages of cells in the subG_1_, G_0_/G_1_ and S/M/G_2_ DNA content are shown. (**F**) Morphological changes and altered expressions of E-cadherin and *β*-catenin in the *BRG1* siRNA-transfected DLD-1 cells.

To examine the biological function of BRG1 in CRC cells, we collected five CRC cell lines and examined the protein levels of BRG1 and BRM. DLD-1, SW480 and HCT116 cells showed high levels of BRG1 and BRM expression, whereas LoVo and SW620 cells expressed neither BRG1 nor BRM (Figure 2A). Nuclear localisation of both BRG1 and BRM in DLD-1 cells are shown in Figure 2B. Then, we performed an RNA interference of *BRG1* to assess the impact of silencing BRG1 on cell proliferation and morphology. The *BRG1* siRNA specifically decreased BRG1 expression; however, there was no significant effect on BRM levels (Figure 2C). Transduction of *BRG1* siRNA significantly reduced cell growth (*P*<0.05; Student’ *t*-test; Figure 2D) and increased the population of cells in the G_1_/G_0_ phase (Figure 2E). As transduction of *BRG1* siRNA did not increase the population of cells in the subG_1_ DNA content, we considered that knockdown of BRG1 did not influence cell viability (Figure 2E). Also, BRG1 knockdown caused the morphologic changes: although cells treated with the negative control siRNA transfectant formed stable cell-to-cell junctions, and E-cadherin and *β*-catenin were linearly localised at the cell–cell borders, the distribution of these molecules was disrupted by the *BRG1* siRNA transfectant (Figure 2F).

### Knockdown of BRG1 upregulates the *PTEN* mRNA levels

**Figure 3:**
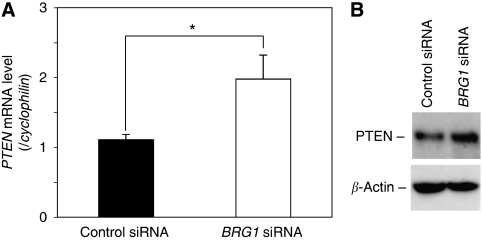
Knockdown of BRG1 upregulates PTEN levels in DLD-1 cells. (**A**) Results of real-time quantitative RT–PCR analysis. The *cyclophilin* mRNA levels were examined as a quality and quantity of control of mRNA. ^*^P<0.05, Student's *t*-test. (**B**) Increased levels of PTEN protein in the *BRG1* siRNA transfectant. *β*-Actin was used as a loading control.

To explore the genes affected by BRG1 that are attributed to elevated cell growth, we performed a cDNA microarray gene expression profile using cDNAs from DLD-1 cells in the presence or absence of the BRG1 knockdown. We confirmed an approximately eight-fold reduction of *BRG1* transcripts by transduction of *BRG1* siRNA in the cDNA microarray experiment (data not shown). Then, we extracted a total of 12 cell cycle-related genes whose expressions were up- or down-regulated ⩾3-fold by transduction of *BRG1* siRNA (Table 1). According to the results, we hypothesised that *PTEN* might have an important role as a downstream target of BRG1 in human CRC, because PTEN was a key tumour suppressor by suppressing the PI3K–Akt signalling pathway in a variety of human cancers (Li *et al*, 1997; Steck *et al*, 1997; Semba *et al*, 2009). Upregulation of PTEN expression was confirmed in DLD-1 (Figure 3) and SW480 cells (Supplementary Data S1). We investigated the altered expressions of the PI3K–Akt signalling-related genes. Although there was no significant difference in the *PIK3CA*, *PIK3CB*, *PIK3CD* and *PIK3CG* genes, decreased levels of gene expression of *PDK1* and *PI3KD1* transcripts were detected by transduction of *BRG1* siRNA (Supplementary Data S2). Furthermore, no change of the Rb and p53 mRNA and protein levels were found in the *BRG1* siRNA-treated DLD-1 cells (data not shown).

### Knockdown of BRG1 downregulates the cyclin D1 expression levels via inhibition of the PI3K–Akt signalling pathway

**Figure 4:**
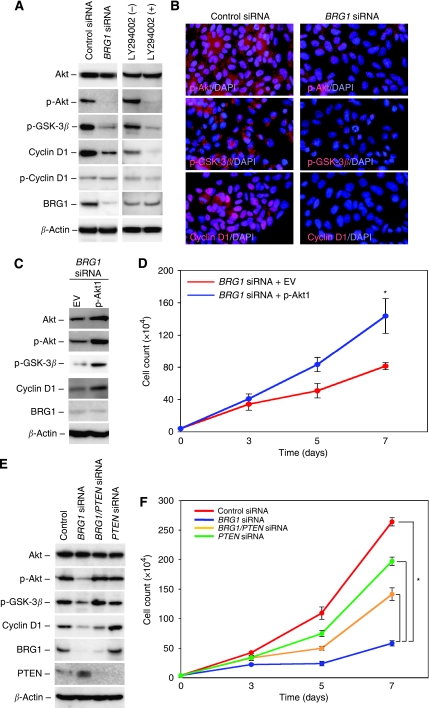
The impact of BRG1 on the cyclin D1 levels via the PI3K–Akt signalling pathway in DLD-1 cells. (**A**) Results of western blot analysis. Cells were also treated with PI3K inhibitor LY294002. *β*-Actin was used as a loading control. (**B**) The status of p-Akt, p-GSK-3*β* and cyclin D1 expressions in the *BRG1* siRNA transfectant. (**C**) Transduction of recombinant Akt expression vector (p-Akt1) inhibited tumour-suppressive effects of *BRG1* siRNA. *β*-Actin was used as a loading control. (**D**) Results of the growth test. ^*^P<0.05, Student's *t*-test. (**E**) Co-transduction of *BRG1* siRNA and *PTEN* siRNA. *β*-Actin was used as a loading control. (**F**) Results of the growth test. ^*^P<0.05, Student's *t*-test.

The major role of PTEN is to suppress tumourigenesis as a negative regulator of the PI3K–AKT signalling pathway (Franke *et al*, 1997). We examined altered expression levels of the key proteins involved in the PI3K–Akt signalling pathway when the cells were transfected with *BRG1* siRNA. Silencing of BRG1 remarkably reduced not only the phosphorylated forms of Akt and GSK-3*β* levels, but also the total amount of cyclin D1 protein levels in DLD-1 cells, whereas transduction of *BRG1* siRNA did not significantly decrease p-cyclin D1 levels (Figure 4A and B). Treatment by the LY294002 PI3K inhibitor showed the same effects on the reduced levels of p-Akt, p-GSK-3*β* and cyclin D1 expression (Figure 4A). The similar effect of silencing of BRG1 on the negative regulation of the PI3K–Akt signalling pathway was confirmed in SW480 cells (Supplementary Data S1). GSK-3*β*-induced nuclear accumulation of *β*-catenin is another main pathway targeting cyclin D1 by upregulation of *cyclin D1* mRNA transcripts (Shtutman *et al*, 1999; Tetsu and McCormick, 1999). Therefore, we examined the amount of *β*-catenin in subcellular fractions and the *cyclin D1* mRNA levels in the *BRG1* siRNA- and control siRNA-transfected cells; however, we did not detect any significant changes in either experiment (Supplementary Data S3). Furthermore, co-transfection of *BRG1* siRNA and p-Akt1 restored cell growth accompanied by upregulation of p-Akt, p-GSK-3*β* and cyclin D1 levels (Figure 4C and D), whereas transduction of *PTEN* siRNA decreased tumour-suppressing effect of BRG1 silencing (Figure 4E and F).

### High levels of BRG1 are associated with the cyclin D1 status in human CRC tissues

**Figure 5:**
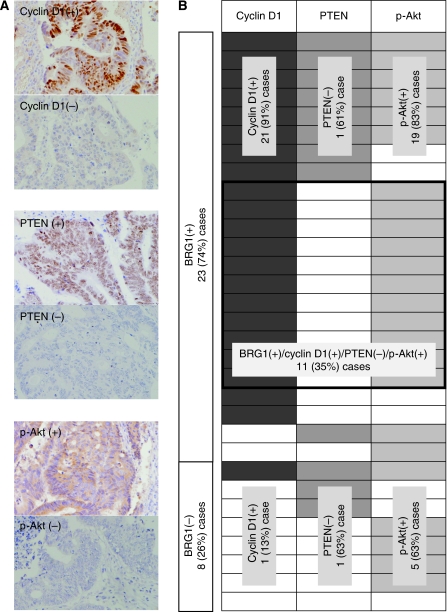
The status of BRG1 expression correlates with high cyclin D1 levels in CRC cases. (**A**) Representative illustrations of immunoreactivities against cyclin D1, PTEN and p-Akt antibodies. (**B**) Summary of immunohistochemical analyses. Immunoreactivities against BRG1, cyclin D1, PTEN and p-Akt were evaluated as described in the text.

Finally, we investigated whether the expression pattern of BRG1 was consistent with that of cyclin D1 expression in human CRC tissues (Figure 5A). The results are summarised in Figure 5B. Of 31 CRCs, 23 (74%) cases showed positive immunoreactivity against BRG1. Interestingly, positive immunoreactivity against cyclin D1 was frequently detected in 21 (91%) of 23 BRG1(+) CRCs and only in 1 (13%) of 8 BRG(−) CRCs. As for the status of PTEN and p-Akt levels, there was no significant difference between BRG1(+) and BRG1(−) CRCs. Overall, 11 (35%) of BRG1(+)/PETN(−)/p-Akt(+)/cyclin D1(+) CRC cases were detected, which supported the results obtained in *in vitro* experiments.

## Discussion

In this study, we examined the biological significance of BRG1, the SWI/SNF chromatin-remodelling factor, during the development of CRC. Our results showed that BRG1 expression was frequently elevated in CRC tissues and that BRG1 knockdown in DLD-1 cells reduced cell proliferation by suppressing the activity of the PI3K–Akt signalling pathway by induction of *PTEN* expression and resultant downregulation of cyclin D1 expression. Interestingly, this correlation between BRG1 and cyclin D1 expression was also observed in human CRC tissues. To our knowledge, this is the first report indicating that aberrant BRG1 expression may promote tumour development and growth through the PI3K–Akt pathway in CRC. In these clinical specimens of human CRC, we found significantly increased expression of BRG1 but not BRM compared with the normal mucosa. In addition, the average ratio of BRG1(+) cells in adenoma and CRC was significantly higher than that in normal mucosa. This positive correlation between BRG1 expression and tissue malignancy was in agreement with the results of gastric cancer (Sentani *et al*, 2001), prostate cancer (Sun *et al*, 2007) and cutaneous melanoma (Lin *et al*, 2010) but not with those of lung cancer (Reisman *et al*, 2003).

In our BRG1 knockdown experiments, we found remarkable reduction of cell proliferation and cessation of cell cycle. As a molecular mechanism of BRG1 affecting cell proliferation, we provided evidence that BRG1 possibly suppressed PTEN expression at the mRNA and protein levels and then downregulated the PI3K–Akt signalling pathway. As reported previously, loss of PTEN (Frattini *et al*, 2005; Sawai *et al*, 2008) and phosphorylation of Akt (Itoh *et al*, 2002) are essential for the tumour progression of CRC cells. Although *PTEN* transcription is regulated by several molecules, including Egr-1 (Virolle *et al*, 2001), c-Jun (Hettinger *et al*, 2007), Id-1 (Lee *et al*, 2009) and TGF-*β* (Chow *et al*, 2010), little is known about the gene regulating *PTEN* transcription during CRC development. Our results may offer a key to understanding the regulatory basis for the *PTEN* mRNA in CRC. Although BRG1 contributed to tumour suppression by interaction with Rb (Dunaief *et al*, 1994; Strober *et al*, 1996; Dahiya *et al*, 2000) and p53 (Bochar *et al*, 2000), other BRG1-related mechanisms may exist for the promotion of tumour growth in CRC. Here, we have proposed the possibility that the transcription of *PTEN* is regulated by BRG1; further study is required to determine what mechanism BRG1 uses for transcriptional regulation of *PTEN*, whether by the disruption of histone-DNA contacts by ATP-dependent chromatin remodellers, or by histone tail modifications including methylation and acetylation or by direct regulation.

Cyclin D1 is an important target of the PI3K–Akt signalling pathway, and overexpression of cyclin D1 may be a significant predictor of CRC progression (Ogino *et al*, 2009). Nuclear *β*-catenin interacts with DNA-binding proteins of the TCF/LEF family and acts as a transcriptional activator of many target genes including cyclin D1 (Shtutman *et al*, 1999; Tetsu and McCormick, 1999). Despite previous reports showing that *β*-catenin expression in the nuclei can be a prognostic marker in CRC patients (Horst *et al*, 2008), and that BRG1 directly interacts with *β*-catenin to promote target gene activation (Barker *et al*, 2001), our results show that BRG1 expression is significantly associated with cyclin D1 expression but not nuclear *β*-catenin expression. This finding derives from our experiments using DLD-1 cells: BRG1 knockdown downregulated expression of cyclin D1 without subcellular change of *β*-catenin (Supplementary Data S3), and p-cyclin D1 expression level was relatively higher than total cyclin D1 levels (Figure 4A). Therefore, it seems reasonable that the aberrant expression of BRG1 can regulate the degradation of cyclin D1 via direct phosphorylation by GSK-3*β* (Diehl *et al*, 1998), but not via nuclear *β*-catenin accumulation.

The data from our cDNA microarray study using *BRG1* siRNA- and negative control siRNA-treated cells suggests the further possibility that BRG1 is involved in regulating other tumour-related genes as well as the *PTEN* gene. In genes upregulated by *BRG1* siRNA transduction, the *regulator of G-protein signalling 2 (RGS2)* gene is suggested to have an important role as tumour suppressor in several human cancers (Cao *et al*, 2006; Smalley *et al*, 2007). The *zipper sterile-α-motif kinase (ZAK)* gene might possess tumour-suppressive activity via the ERK and JNK pathways in CRC development as well as in lung cancer (Yang *et al*, 2010). In addition, recent studies have shown that *Annexin1 (ANXA1)*, which was listed as a gene downregulated by BRG1 knockdown, regulates TGF-*β* signalling and promotes metastasis formation (de Graauw *et al*, 2010); loss of ANXA1 was associated with tumour progression in human breast cancer (Shen *et al*, 2006). In this study, we provide novel evidence that BRG1 suppresses tumour growth via *PTEN* transcription; however, it is possible that PTEN cooperates with the other genes mentioned above. Additional study of the genes potentially regulated by BRG1 could uncover a multifaceted role of BRG1 in CRC development.

## Figures and Tables

**Table 1:** Cell cycle-related genes differentially expressed in DLD-1 cells transfected with *BRG1* small interfering RNA (siRNA) and negative control siRNA

**Accession number**	**Gene symbol**	**Gene title**	**Fold change**
*Upregulated genes*			
NM_018571	*ALS2CR2*	Amyotrophic lateral sclerosis 2 chromosome region, candidate 2	3.52
NM_002923	*RGS2*	Regulator of G protein signalling	3.23
NM_000314	*PTEN*	Phosphatase and tensin homolog	3.22
NM_078469	*BCCIP*	BRCA2 and CDKN1A interacting protein	3.20
NM_133646	*ZAK*	Sterile-α-motif and leucine zipper containing kinase	3.00
			
*Downregulated genes*			
AB013462	*FZR1*	mRNA for Fzr1	0.29
NM_001924	*GADD45A*	Growth arrest and DNA-damage-inducible, α	0.26
NM_000389	*CDKN1A*	Cyclin-dependent kinase inhibitor 1A	0.25
NM_004083	*DDIT3*	DNA-damage-inducible transcript 3	0.23
NM_005427	*TP73*	Tumour protein p73	0.21
NM_012191	*NAT6*	N-acetyltransferase 6	0.21
NM_000700	*ANXA1*	Annexin A1	0.17
