# Supplementary material for: Regulation of PTEN expression by the SWI/SNF chromatin-remodelling protein BRG1 in human colorectal carcinoma cells
Source: Br J Cancer. 2010 Nov 23;104(1):146–54. doi: 10.1038/sj.bjc.6606018 (PMC3039810; doi:10.1038/sj.bjc.6606018)
Supplement: Supplementary data S1–S3 [file 6606018x1.ppt]

## Slide 1
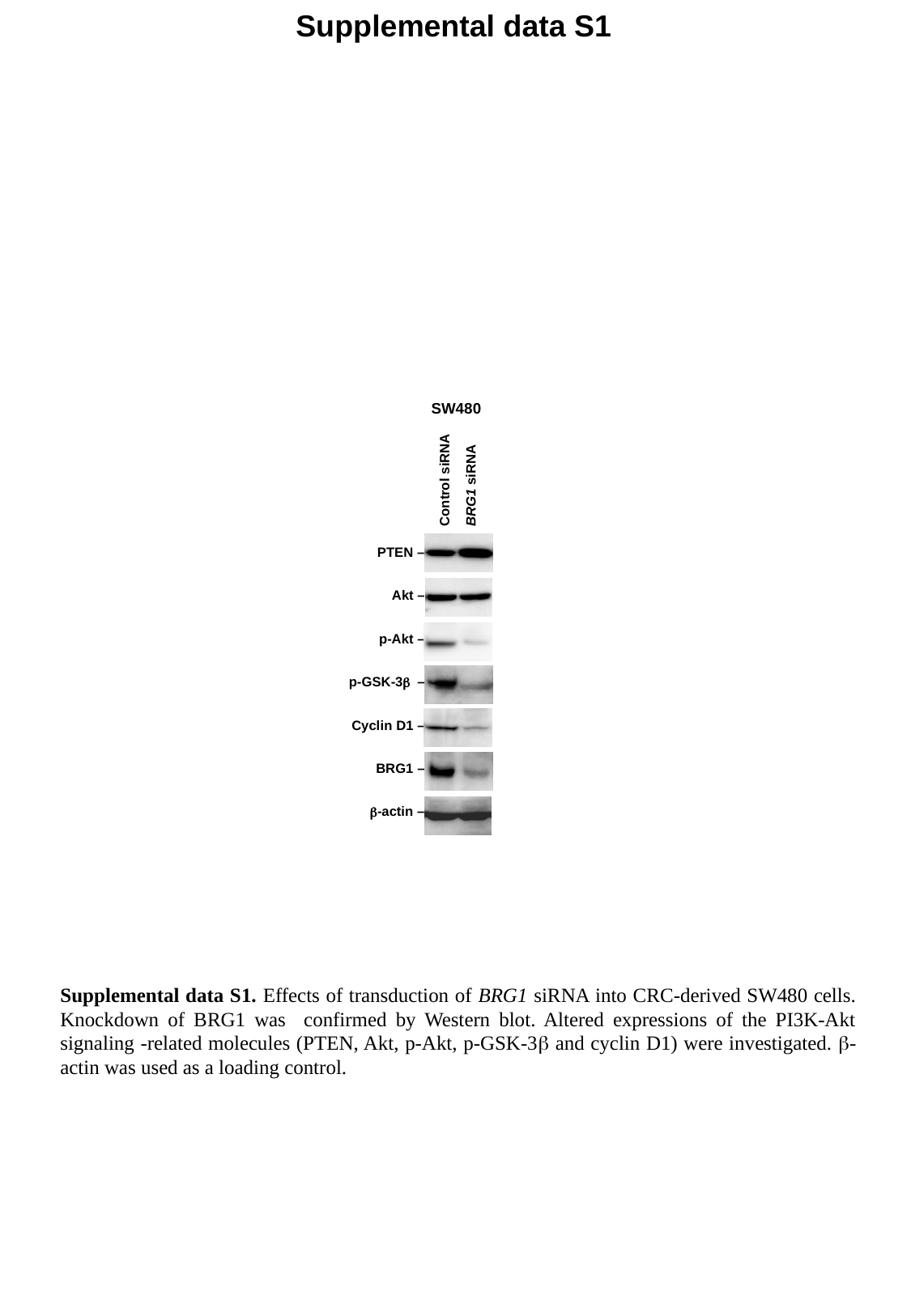

Supplemental data S1
SW480
Control siRNA
BRG1 siRNA
PTEN –
Akt –
p-Akt –
p-GSK-3β –
Cyclin D1 –
BRG1 –
β-actin –
Supplemental data S1. Effects of transduction of BRG1 siRNA into CRC-derived SW480 cells. Knockdown of BRG1 was confirmed by Western blot. Altered expressions of the PI3K-Akt signaling -related molecules (PTEN, Akt, p-Akt, p-GSK-3β and cyclin D1) were investigated. β-actin was used as a loading control.

## Slide 2
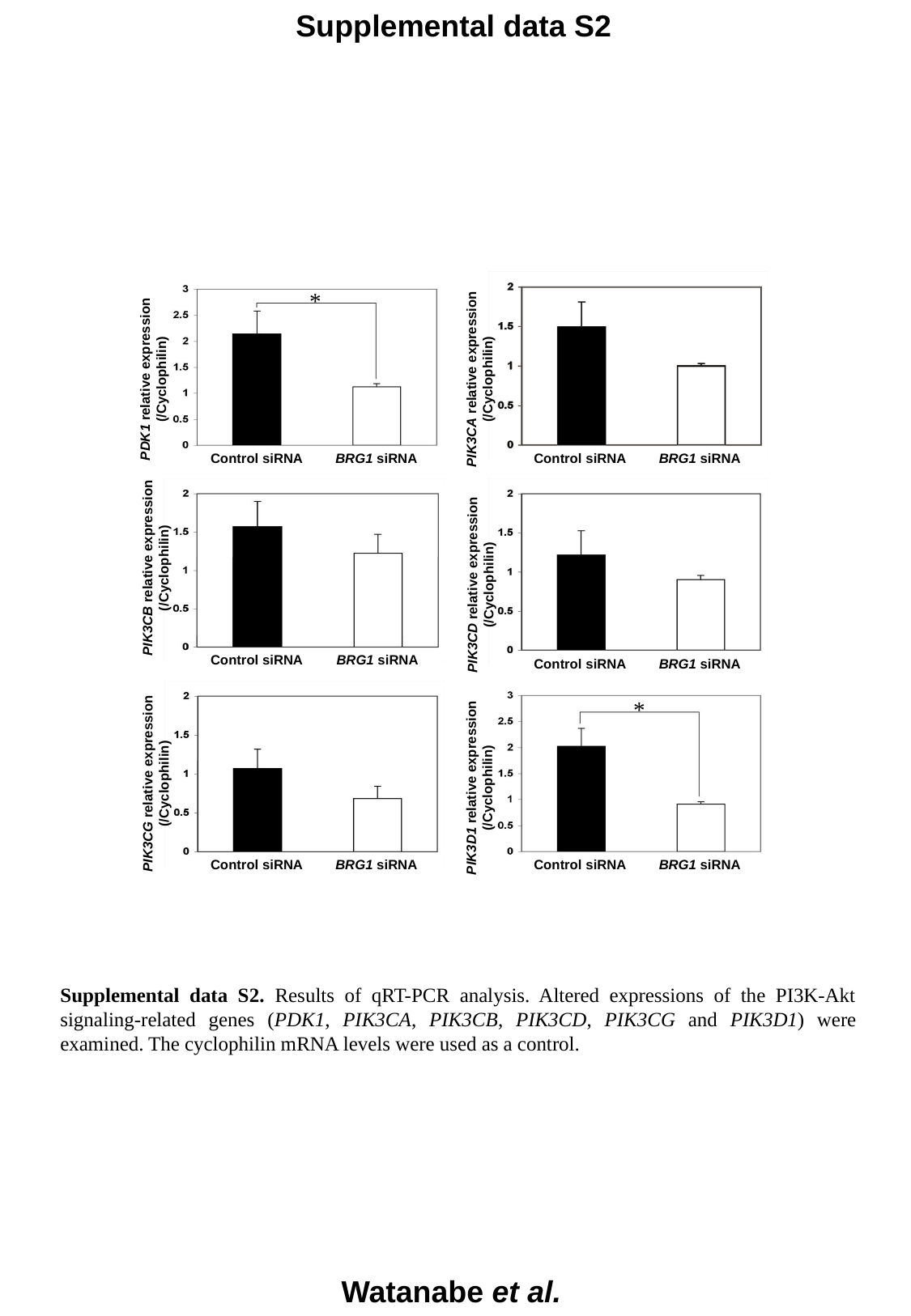

Supplemental data S2
PIK3CA relative expression
(/Cyclophilin)
Control siRNA
BRG1 siRNA
*
PDK1 relative expression
(/Cyclophilin)
Control siRNA
BRG1 siRNA
PIK3CB relative expression
(/Cyclophilin)
Control siRNA
BRG1 siRNA
PIK3CD relative expression
(/Cyclophilin)
Control siRNA
BRG1 siRNA
PIK3CG relative expression
(/Cyclophilin)
Control siRNA
BRG1 siRNA
*
PIK3D1 relative expression
(/Cyclophilin)
Control siRNA
BRG1 siRNA
Supplemental data S2. Results of qRT-PCR analysis. Altered expressions of the PI3K-Akt signaling-related genes (PDK1, PIK3CA, PIK3CB, PIK3CD, PIK3CG and PIK3D1) were examined. The cyclophilin mRNA levels were used as a control.
Watanabe et al.

## Slide 3
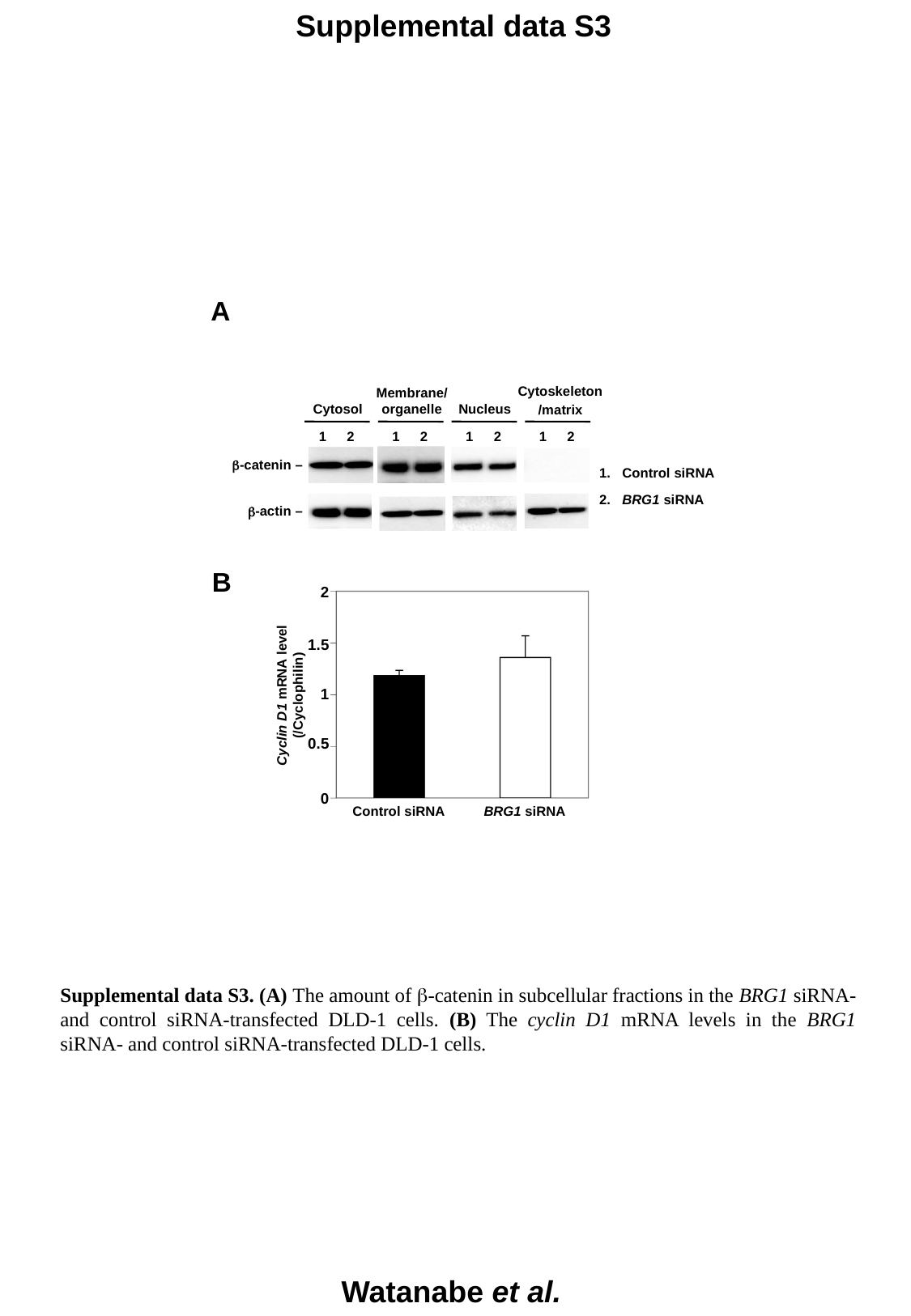

Supplemental data S3
A
Membrane/organelle
Cytoskeleton
/matrix
Cytosol
Nucleus
1
2
1
2
1
2
1
2
β-catenin –
β-actin –
1. Control siRNA
2. BRG1 siRNA
B
2
1.5
Cyclin D1 mRNA level
(/Cyclophilin)
1
0.5
0
Control siRNA
BRG1 siRNA
Supplemental data S3. (A) The amount of β-catenin in subcellular fractions in the BRG1 siRNA- and control siRNA-transfected DLD-1 cells. (B) The cyclin D1 mRNA levels in the BRG1 siRNA- and control siRNA-transfected DLD-1 cells.
Watanabe et al.
